# Diagnostic testing holds the key to NTD elimination

**DOI:** 10.1371/journal.pntd.0009626

**Published:** 2021-08-19

**Authors:** Catharina Boehme, Sergio Carmona, Sarah Nogaro, Mwelecele Malecela

**Affiliations:** 1 FIND, Geneva, Switzerland; 2 Department of Control of Neglected Tropical Diseases, World Health Organization, Geneva, Switzerland; Swiss Tropical and Public Health Institute, SWITZERLAND

## Abstract

“Fit-for-purpose” diagnostic tests have emerged as a prerequisite to achieving global targets for the prevention, control, elimination, and eradication of neglected tropical diseases (NTDs), as highlighted by the World Health Organization’s (WHO) new roadmap. There is an urgent need for the development of new tools for those diseases for which no diagnostics currently exist and for improvement of existing diagnostics for the remaining diseases. Yet, efforts to achieve this, and other crosscutting ambitions, are fragmented, and the burden of these 20 debilitating diseases immense. Compounded by the Coronavirus Disease 2019 (COVID-19) pandemic, programmatic interruptions, systemic weaknesses, limited investment, and poor commercial viability undermine global efforts—with a lack of coordination between partners, leading to the duplication and potential waste of scant resources. Recognizing the pivotal role of diagnostic testing and the ambition of WHO, to move forward, we must create an ecosystem that prioritizes country-level action, collaboration, creativity, and commitment to new levels of visibility. Only then can we start to accelerate progress and make new gains that move the world closer to the end of NTDs.

Ahead of the second-ever World Neglected Tropical Disease (NTD) Day in January 2021, and amid the global Coronavirus Disease 2019 (COVID-19) crisis, the World Health Organization (WHO) launched a new roadmap for the prevention, control, elimination, and eradication of NTDs—a group of 20 diseases affecting more than one billion people worldwide [1]. Diagnostic testing is central to safeguarding decades of progress in NTDs and must be strategically leveraged to reach the goals laid out in the new NTD roadmap.

Stepping back, we recognize the massive progress that has been made to combat NTDs. Today, 500 million fewer people need treatment for these debilitating diseases than in 2010, and 40 countries or areas have eliminated at least one of the 20 [1]. Yet, despite these gains, NTDs continue to impose a devastating human, social, and economic toll on the world’s poorest and most vulnerable communities [2–6]. COVID-19 is compounding the situation by wreaking havoc on health systems, which impacts progress on NTDs: this includes interruptions to mass treatment campaigns for diseases controlled through preventive chemotherapy (PCT) or individual case management interventions, as well as rerouting the already sparse available funding and resources [7].

Diagnostic testing has been central to the COVID-19 response even with the introduction of vaccines. The rapid ramp up of research and development (R&D), the scaling up of low-cost and decentralized testing, and country-led approaches to tailored testing strategies for COVID-19, as well as lessons learned, can also provide new thinking around testing for NTDs. The new NTD roadmap offers a series of multisectoral actions and intensified, cross-cutting approaches to get us back on track—with diagnostics central to unlocking and accelerating this progress [1].

However, the NTD roadmap shows that, of all 20 diseases or disease groups, just 2 (yaws and snakebite envenoming) are supported by adequate and accessible diagnostic tools. Six have no diagnostic tests available at all, with tools for each of the remaining conditions in urgent need of adaptation, modification, and/or improved accessibility (likely a more cost-effective option than the development of new diagnostics for these NTDs) [1]. This has to change. NTDs cannot continue to be neglected in favor of other competing priorities, or we risk losing the progress made to date.

Until the COVID-19 pandemic thrust testing into the spotlight, diagnostics have been a “silent partner” in healthcare, receiving little by way of international attention and funding, specific country strategies, and dedicated budget lines. NTDs are no exception. Just 5% of the (limited) funding made available to NTDs has been invested in new diagnostics, compared with 44% and 39% on basic research and medicines and vaccines, respectively [1]. For most NTDs, diagnostics are a market failure situation, and as such, are not commercially viable enough to attract private investment. Consequently, very few diagnostic developers engage in this area—contrary, for example, to COVID-19, where developers are in the hundreds. Furthermore, as some diseases approach the last mile of elimination, falling infection rates precipitate the need for increasingly sensitive tests [1]. But progress in R&D is slow and fragmented, with a lack of engagement and coordination between governments, industry, donors, and development actors, leading to the duplication—and potential waste—of scant resources. While serial testing using multiple diagnostic tools or techniques can compensate for low sensitivity [8], such approaches are associated with increased costs of testing, sample collection, and transportation.

Closing the diagnostic gap then, is a prerequisite to achieving the global ambition for NTDs, with the new NTD roadmap giving a blueprint for action. It is for this reason that we call on governments, industry, donors, and development actors to

- Prioritize country-level diagnostic action: As we enter a new era in NTD management and control, we need to shift from traditional, donor-led models to country-driven initiatives. Government ministries must engage with, and advocate on behalf of, their poorest and most vulnerable populations so that no one is left behind. Political frameworks should prioritize diagnostics for NTDs in line with local disease burdens, and as part of fully funded, national health action plans that include a commitment to seeing the process through. Capacity building for diagnostics is also essential at country, sub-regional, and regional levels, including the establishment of laboratory networks, so that testing can be implemented in field settings.
- Collaborate and create: There is never going to be a one-size-fits-all for NTD diagnostics. If targets are to be achieved, we need global frameworks that enable industry, manufacturers, and pharmaceutical companies to engage in the whole process, from R&D to supply chain logistics. Companies need to share knowledge, learnings, and innovation across multiple diseases. This will mean breaking silos and finding new ways to harness the power of existing products, technologies, and infrastructures. Further, it will mean creating economies of scale through regional manufacturing hubs and finding new, cross-cutting approaches to drive systemic change. To obtain the maximum access to technology and relevant intellectual property rights for NTD diagnostics, it is important to ensure that such rights are broadly available (non-exclusively) in NTD-endemic countries and are affordable (e.g., zero royalty rights).
- Commit to new levels of visibility: The resources needed to realize that this ambition is limited, with a lack of visibility around the diagnostic landscape undermining progress in NTD management and control. Creating an ecosystem with visibility, transparency, and integration at its core will help streamline programmatic action, reduce the risk of duplication, and leverage the full potential from this limited pool. To do this, industry, donors, and other development actors must provide the information needed to map both funding and product landscapes. Using this information to create a virtual product pipeline will bring an unprecedented level of transparency to diagnostic developments—harmonizing multisectoral efforts and creating a robust information platform from which new collaborations, synergies, and innovation can grow. Developing an online open-access diagnostic pipeline for WHO NTD roadmap priority pathogens would serve multiple purposes: (i) drive advocacy to address critical product and funding gaps; and (ii) reduce the likelihood of duplication of efforts. Together, this would strengthen partnerships across all stakeholders, from donors to industry partners, to accelerate development, evaluation, and adoption of diagnostic solutions for NTDs. The newly established NTD Diagnostic Technical Advisory Group (DTAG) to WHO NTD department has already identified the priority diagnostic needs for NTD programs not only in terms of developing new tools, but also the accessibility of existing tools [9]. Several sub-groups that focus more narrowly on single diseases or specific topics (i.e., skin NTDs or cross-cutting) have been established and have been tasked to develop tool and biomarker agnostic target product profiles (TPPs), which are now available (for the most part) on WHO website for use by any diagnostic manufacturer to support development of their specific technology. Alignment with these diagnostic priorities by all stakeholders is strongly recommended to facilitate attainment of WHO 2030 NTD roadmap goals.

- Establish NTD biobanks: Biobanks are required for the clinical evaluation and validation of new diagnostic tests. Establishing local biobanks would support a country-driven approach as well as allowing for head-to-head comparisons between tests and assessments of cross-reactivity across different NTDs.
- Invest in existing diagnostics: The development of new diagnostics is a complex process, and the time from development to implementation can be lengthy. Training laboratory staff in the use of existing diagnostics and the establishment of robust quality control systems are effective approaches to achieving shorter-term improvements.

There is a long road ahead, but the past 10 years have shown us what can be achieved when governments, industry, donors, and development actors are bound by a shared, global goal. As we look forward to the next decade, we must prioritize country-level action, collaboration, creativity, and commitment to new levels of visibility, if we are to finally end the neglect of NTDs.
